# Pressure Algometry Validation and Determination of Efficacy of Articaine Hydrochloride Ring Block in Antler Removal in Red Deer (*Cervus elaphus*)

**DOI:** 10.3390/ani10112023

**Published:** 2020-11-03

**Authors:** Farzin Sahebjam, Kavitha Kongara, John Paul Chambers, Ruth Ellen Walker, Rafea Naffa, Nicolas Lopez-Villalobos, Preet Mohinder Singh

**Affiliations:** 1 Animal Welfare Science and Bioethics Centre, School of Veterinary Science, Massey University, Palmerston North 4474, New Zealand; k.kongara@massey.ac.nz (K.K.); J.P.Chambers@massey.ac.nz (J.P.C.); P.M.Singh@massey.ac.nz (P.M.S.); 2 School of Veterinary Science, Massey University, Palmerston North 4474, New Zealand; ruth.walker1@outlook.com; 3 New Zealand Leather and Shoe Research Association, Palmerston North 4414, New Zealand; rafea.naffa@lasra.co.nz; 4 School of Agriculture and Environment, Massey University, Palmerston North 4474, New Zealand; N.Lopez-Villalobos@massey.ac.nz

**Keywords:** algometry, articaine hydrochloride, deer, local anaesthetic, mechanical nociceptive threshold, pain

## Abstract

**Simple Summary:**

Red deer (*Cervus elaphus*) are farmed in New Zealand for the production of velvet antler. Velvet is harvested as living antler tissue, and currently lignocaine is the only licensed local anaesthetic approved for deer antler removal in New Zealand. The use of lignocaine is not without consequences, including drug residues in harvested velvet antler tissue and its short acting analgesic effect. This study was designed to determine the effect of local anaesthetic, articaine hydrochloride as an alternative treatment and to establish the baseline measurements of mechanical nociceptive threshold in 40 male yearling red deer. Ten of the forty enrolled deer were selected for the articaine efficacy study. The mechanical nociceptive thresholds were measured using a handheld algometer applied at 4 points; the cranial, medial, caudal and lateral aspects of the base of each antler. The force applied, which resulted in a movement by the animal, was recorded in newtons (N). This study showed that nociceptive threshold response in deer could be reliably measured, and articaine proved to be a promising alternative for velveting the deer antlers.

**Abstract:**

New Zealand deer farming centres on the production of meat and velvet antler. Velvet antler removal is a painful procedure and currently, New Zealand Animal Welfare regulations dictate surgical removal of velvet antlers under lignocaine anaesthesia. To improve our knowledge on the efficacy and duration of other local anaesthetics to mitigate pain after antler removal, it is important to accurately assess and quantify pain arising from antler removal. Therefore, the current study was designed to validate mechanical nociceptive threshold (MNT) testing using a Wagner hand-held algometer, and to apply this methodology to assess the efficacy and duration of action of articaine for antler removal in deer. Baseline force (N) required to elicit the nociceptive response was recorded in 40 yearling male red deer on three alternate days. Ten of the 40 animals were selected for antler removal after administration of 4% articaine hydrochloride as a ring block. The duration of analgesic efficacy of articaine was assessed by algometry across 5 time points. There was a significant difference in MNTs among the three days (day 3 versus day 1 (*p* < 0.0001), day 2 versus day 1 (*p* < 0.0001), and day 1 versus day 2 (*p* < 0.01)). Positive correlations were observed between weight, antler length and thresholds. The MNT values remained above 20N for 6 h after removal of velvet antlers under the articaine ring block. This study provides valuable information about the use of MNT in red deer. These findings lay a foundation for future studies in the topics of peri-operative and postoperative pain management in deer antler removal, and a possible alternative use for articaine.

## 1. Introduction

Pain assessment related to deer antler removal was assessed by both behavioural and physiological measurements [1]. The interpretation of painful behaviour is difficult when deer are removed from their normal environment and their social grouping [2]. Most physiological parameters assess stress, which is attributed to pain, but handling stress and restraint can effect behaviour and pain assessments [2]. There were no differences in cortisol levels in the deer, which had antlers removed with analgesics compared to those removed without any analgesia [3], probably due to handling stress or isolation during the procedure [3]. Physiological parameters such as heart rate can be an indicator of stress; it increased following antler removal [4] but was found to be an unreliable measurement for pain assessment. There was no difference observed in the behaviour between stags that had antlers removed and those left intact [5]. In the same study, the authors observed increased walking as a result of both antler removal and restraint only. In other studies, a significant difference in behaviour was demonstrated in deer after antler removal with and without analgesia [3,6]. Deer, being a prey species, evolved to not exhibit the visible signs of pain [7].

Mechanical nociceptive threshold testing (MNT) or algometry is used extensively for assessment of pain and analgesia in animals. Sheep in pain had lower mechanical nociceptive thresholds as compared to healthy animals [8], which aligned with the findings in sheep with footrot [9]. Pressure algometry was successful in detecting and screening on-farm mastitis [10] and lameness in dairy cows [11]. MNT scores at the tail stump were lower in cows with docked tails, as compared to undocked tails, suggesting hyperalgesia [12]. Janczak et al. (2012) performed an extensive study on validation of MNT in piglets and suggested its use for testing analgesic drugs for surgical procedures in pigs, but only after an acclimatisation period of 2 to 3 weeks of repeated testing in normal subjects [13]. Subsequently, MNT was used in testing the efficacy of various analgesic drugs for treating lameness in experimentally induced lameness in sows [14,15]. All of the above-mentioned studies demonstrate the reliability of algometry as a method to objectively measure mechanical nociception. 

The Wagner hand-held algometer is a commercially available mechanical nociceptive threshold testing device that was validated for use in various species. The analgesic efficacy of meloxicam for calf dehorning was tested using a Wagner FPX50™ algometry device (Wagner Instruments, Riverside, CT, USA), with significantly increased pain thresholds recorded in calves receiving meloxicam treatment, as compared to the control group [16]. The comparison of analgesia provided by topical anaesthetic spray (Tri-Solfen, Bayer Animal Health, Pymble, NSW, Australia) versus meloxicam administered subcutaneously, for calf disbudded with and without xylazine sedation, was investigated by using the Wagner FPX50™ device. MNT assessment was recorded in 4 different sites around each horn bud from the first hour, until 24 h after disbudding [17]. They reported a significant increase in nociceptive thresholds in calves under sedation, following treatment with meloxicam or Tri-solfen, as compared to the unsedated calves [17]. The same device was also validated and used in horses [18] and dogs [19].

Currently lignocaine hydrochloride is the only licensed local anaesthetic for deer in New Zealand, which is being used as a ring block around the antlers, before the surgical removal of velvet antlers [20,21]. Velvet antler is mainly consumed by people for its medicinal effects [22]. Previously, Woodbury et al. showed concerns about the presence of lignocaine in antler tissue [23]; similarly, Nelson et al. stated the presence of lignocaine metabolites called 2,6-dimethylaniline (DMA), after consumption of velvet antlers in people [24], which is considered to be carcinogenic and toxic [25]. Therefore, there is a need to evaluate a safer local anaesthetic such as articaine. The pharmacokinetics and efficacy of articaine hydrochloride was recently studied in red deer after administration of 4% articaine HCL as a ring block around the base of the deer antler [26,27]. Venkatachalam et al. demonstrated a dose of 1 mL/cm around the antler circumference, at the region of the pedicle, was sufficient to induce analgesia in 3–5-min in red deer. Articaine rapidly metabolises to its inactive metabolites by plasma esterases, thus making it less toxic, as compared to lignocaine or bupivacaine [26,28]. Rapid pain relief, fast elimination, and minimal residues in the antlers, suggests that articaine hydrochloride would be the best candidate for local anaesthesia use in red deer antlers, in the future.

The objective of this study was to establish a mechanical nociceptive threshold testing technique using a hand-held algometer in farmed red deer (*Cervus elaphus*). The validation results would enable the use of an algometer in testing the efficacy of analgesic and local anaesthetic drugs such as articaine hydrochloride, for antler removal in male deer. In addition, this study aimed to investigate the duration of the analgesic effect after articaine administration, via a ring block, using a hand-held algometer.

## 2. Materials and Methods

### 2.1. Animals

The animal study was approved by the Massey University Animal Ethics Committee (refer to the end of the manuscript), and at the completion of the study, the enrolled deer were returned to the farm pastures for venison/meat production. Forty yearling male deer weighing 116.6 ± 11.38 kg from the Massey University Deer Unit were recruited for validation assessment of algometry and measurement of baseline MNT. Baseline measurements of body weight and antler length were recorded at the start of the study. Deer body weight was calculated in kilograms using the Tru-Test load bars connected to a Tru-Test XR5000 (Datamars Limited, Auckland, New Zealand), while passing through the handling facility. Antler length was measured by placing a stainless-steel metric ruler alongside the medial edge of each antler. The length was recorded in centimetres from the skull-base to the antler tip. Ten out of 40 animals were selected for the assessment of the duration of action for articaine hydrochloride after surgical antler removal. The animals were selected based on appropriate maturation and antler size determined by the market demand. Based on our pilot study, preliminary results, and including the following assumptions; medium effect size of 0.25, number of cases (40 animals), and number of groups and covariates, the statistical priori power was calculated to be above 0.9, by the G-power software program (version 3.1, Heinrich-Heine-Universität Düsseldorf, Düsseldorf, Germany). All animals were clinically inspected by a veterinarian for demeanour, lameness, or any current injuries around the head before the experiment. All deer were kept in paddocks throughout the study period and had free access to pasture and water. On the day of treatment, all deer were mustered into an indoor handling facility on the farm. The deer were placed into holding pens, each containing six deer. Once all deer were inside, two deer were guided carefully from the holding pen to the hydraulic crush (Heenan Work Room, Farmquip, New Zealand). The hydraulic crush enabled the safe handling of deer by confining them between padded walls, with the operator positioned next to the animal. The sides of the crush had curtains above the walls, which could be closed to reduce stimulation and exposure to stressors, while allowing the operator quick and easy access for manipulations on the animals. After each treatment or recording was performed, the deer were released back into a holding pen. While indoors, they had access to water and concentrated feed (Multi-feed nuts, Sharpes Stock Feeds, Carterton, New Zealand). At the completion of each treatment day, all animals were returned to the paddock.

### 2.2. Pressure Algometry

**Figure 1:**
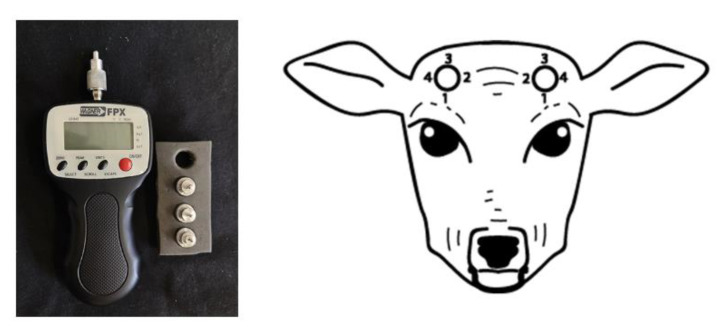
**Left**: Wagner hand-held algometer (FPX 20) with 2 mm diameter steel probe. **Right**: Sites of algometer probe placement around the base of the antler to measure nociceptive thresholds. 1 = cranial, 2 = medial, 3 = caudal and 4 = lateral.

**Figure 2:**
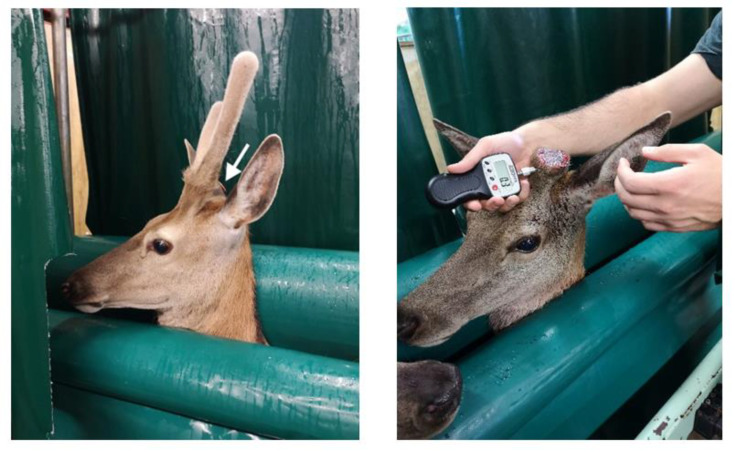
**Left**: Deer yearling showing early antler growth. Note the acute angle between the antler and the poll of the head. The white arrow indicates the site that the hand-held algometer was placed for the MNT measurements. **Right**: Pressure algometry in red deer using Wager^®^ hand-held algometer below the level of velvet antler removal. This picture illustrates the method used to measure force (N) at the cranial aspect of antler.

A hand-held algometer (FPX 25, Wagner Instruments, Greenwich, CT, USA) with a 2 mm-diameter round stainless-steel tip was used to measure the MNT of deer. A single, experienced investigator measured the mechanical nociceptive thresholds of all study animals. For the validation of the hand-held algometer, the MNT measurement was conducted across three alternate days in one week (every second day for 3 test days). The tip of the algometer was placed on cranial, caudal, medial and lateral sites around the base of the antler, 1 cm below the pedicle (the site of antler growth) (Figure 1 and Figure 2). The force was applied (each time for a duration of 1 s by counting 1001) by the operator 1 cm below the pedicle, which corresponded to the antler root at each of the four sites. The behavioural response indicating the attainment of threshold was head shaking and the algometer reading was automatically held at that response. The reading was recorded in newtons (N). Half of the deer were tested on the right antler, followed by the left antler and vice versa. This was performed to minimise the animal’s anticipation of the procedure and reduce bias. The cut-off point for pressure algometry was set at 50 N, to minimise any tissue damage and extra discomfort in animals [8].

### 2.3. Local Anaesthesia with Articaine Hydrochloride

Articaine hydrochloride (99.9%) was purchased from SCI Pharmtech (Taoyuan, Taiwan) and 40 mg/mL, or 4% injectable solution was prepared from standard powder. The dosage was calculated at 1 mL/cm around the antler pedicle circumference. Before any injections, the base of the antlers was measured in centimetre, using a cloth tape to calculate the volume (mL) of the 4% articaine hydrochloride required. Using a 20-gauge needle attached to a 20-mL syringe, articaine hydrochloride (4%) was administered subcutaneously (SC) as a complete ring block (4–5 injections), 2–3 cm below the pedicles for each antler. The baseline readings of the MNT were recorded prior to the injections. Then, 10 min later, MNT was re-measured followed by a nick test (rubbing saw blade gently against the velvet antler) to ensure sufficient analgesia before antler removal. Tourniquets were applied before injections to control excessive bleeding from the major antler vessels around the antlers. Then, MNT assessment was performed 2, 4, and 6 h after the surgical removal of antlers (5 time-points in total). The force reading on the device froze with every sudden head shake (withdrawal response followed by stimulated mechanical pain), and the output on the screen was recorded in a data collection sheet. A single MNT test was performed at each time point. In any case of misalignment of the tip of the algometer to the antler surface, the measurement was repeated. Half of the deer were tested on the right antler first, followed by the left antler, and vice versa. This was performed to minimise the animal’s anticipation of the procedure and reduce confounding factors. Any MNT measurement above 50 N was recorded as 50 N, to minimise any tissue damage and discomfort in the animals.

### 2.4. Statistical Analysis

The statistical analyses were performed in SAS 9.4 (SAS Institute Inc., Cary, NC, USA) and GraphPad Prism 8.3.0 (GraphPad Software Inc., San Diego, CA, USA). The dependent variable, which was force in newtons, was analysed with the MIXED procedure, using a linear mixed model for repeated measures. The Kolmogorov-Smirnov (KS) test indicated that the dependent variable followed a normal distribution, and data were analysed on the nominal scale without a numerical transformation. The model included the fixed effects; day of measurement (1, 2 and 3), antler (right or left), antler site within antler (cranial, medial, caudal and lateral) and as covariates, antler lengths (cm) and deer body weight (kg). The repeated measures on the same deer were modelled with a compound symmetry error structure, which was determined as the most appropriate residual covariance structure, based on Akaike’s information criterion. Least-squares means and standard errors (SEM) for the fixed effects were obtained and used for multiple mean comparisons, using the Fisher’s least significant difference, as implemented in the LSMEANS (Least Squares Means) of the MIXED procedure, with a Bonferroni adjustment. Pearson correlation coefficients among antler lengths (cm), animal body weight (kg) and MNT (N) measurements were obtained using the CORR (Correlation) procedure. Estimates of correlation coefficients with *p* < 0.05 were considered significant from zero. 

## 3. Results

All forty animals enrolled were used in the study, and none of the animals were excluded for injury, discomfort, disease or no response to the nociceptive threshold assessment. For the validation of hand-held algometer, a total of 946 out of 960 force test results from a minimum of 3.1 N to a maximum of 46.4 N were obtained in the threshold assessments. Fourteen recordings out of 960 measurements, measured in four animals, were excluded from data analysis, since they did not respond, and they were recorded as “No Response (NR)” with a reading force of above 50 N, which was the cut-off point. 

All ten animals that were selected for analgesia with articaine hydrochloride (4%), showed no side-effects or reactions followed by the treatments.

### 3.1. Hand-Held Algometer Validation 

The least-squares means (±SEM) for MNT on days 1, 2 and 3 were 15.49 ± 0.55, 14.18 ± 0.55, 18.74 ± 0.55 newtons, respectively. The estimates of variance components were σ_a_^2^ = 8.58, and σ_e_^2^ = 28.55, and total variance = σ_a_^2^ + σ_e_^2^ = 37.13, indicating that variation between the animals was 23.2% of the total variation.

**Figure 3:**
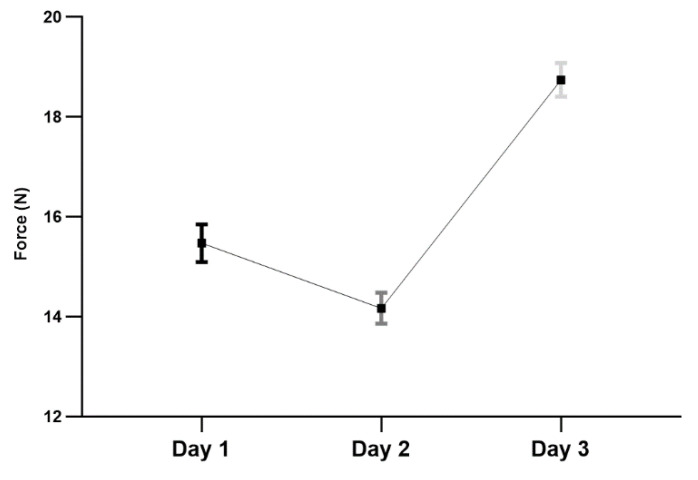
The least-squares means (±SEM) of MNT (N) over three alternate days. The values between all days were significantly different from each other (*p* < 0.01 for Day 1 vs. Day 2, *p* < 0.0001 for Day 1 vs. Day 3, *p* < 0.0001 for Day 2 vs. Day 3).

The results showed that the force measurements in day 3 of the experiment were significantly higher, as compared to days 1 and 2 (*p* < 0.0001 for both). In addition, the forces measured on day 1 were statistically lower, as compared to day 2 (*p* < 0.01). Figure 3 shows the nociceptive thresholds (N) in three different days of experiments. 

### 3.2. MNT Measurements from Left and Right Antlers, and the Testing Sites

The least-squares means (±SEM) of MNTs between the right and left antlers were not significant (right; 16.17 ± 0.52 and left; 16.10 ± 0.52 N). The least-squares means (±SEM) for the cranial, medial, caudal and lateral aspects of the antlers were measured 16.23 ± 0.67, 16.17 ± 0.67, 16.72 ± 0.67 and 15.44 ± 0.67 N, respectively, with no statistical difference. 

### 3.3. The Relationship between Antler Length and Weight with Pain Thresholds in Deer

In general, there was a moderate and positive correlation between body weight (kg) and antler length (cm) with r = 0.6170 and *p* < 0.0001. The results showed a low and positive correlation between antler lengths (cm) and the pain thresholds (r = 0.1580, *p* < 0.001, and r = 0.1025, *p* < 0.05, for right and left antler, respectively). Furthermore, the results showed a low and positive correlation between body weights (kg) and the pain thresholds (N) (r = 0.1746, *p* < 0.0001, and r = 0.1356, *p* < 0.01, for right and left antler, respectively). 

### 3.4. MNT with Articaine Hydrochloride 4% Ring Block

**Figure 4:**
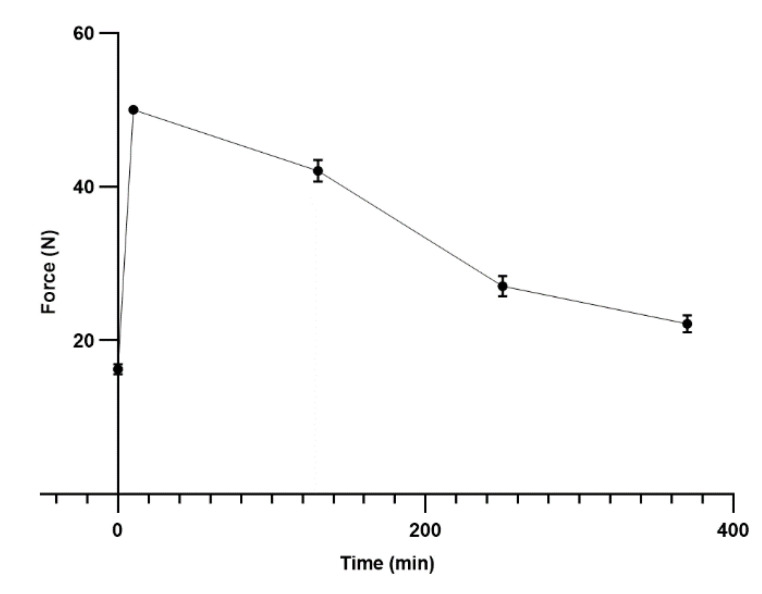
The least-squares means (±SEM) shows the obtained nociceptive thresholds or forces (N) at different time points after ring block with articaine hydrochloride 4% (Each data point represents 10 animals).

The least-squares mean (±SEM) of body weight was measured 125.01 ± 3.20 kg for ten animals, and the MNT values were normally distributed. The least-squares mean (±SEM) of MNT was measured as 26.93 ± 1.56 newtons. The least-squares means at all time-points were significantly higher compared to each other (*p* < 0.0001) and MNT at time 240 was significantly different, as compared to 360 (*p* = 0.0001), suggesting up to 6 h of the effective analgesia in deer antlers after administration of articaine hydrochloride (4%) (Figure 4).

Based on the obtained results in force records, a scaling system was established in our study, which determined; 0.1–20 N as baseline (B) or grade 1, 20–30 N as Low (L) or grade 2, 30–40 N as medium (M) or grade 3, and 40–50 N as High (H) or grade 4. Therefore, the forces measured at time point zero was considered baseline or B, time points 10 min and 2 h were considered high or H, and time points 4 and 6 were considered L or low in force detection scaling (Table 1).

## 4. Discussion

The aim of this study was to validate the use of a hand-held algometer for the measurement of mechanical nociceptive thresholds in deer. Algometry is considered as an objective [29] and a highly repeatable [30] method to quantify pain. Mechanical nociceptive threshold testing or MNT is one of the commonly used technique to assess pain and the efficacy of analgesic drugs in research animals [31,32]. In this study, the least-square means of MNT (±SEM) between right and left antlers, and between the antler sites did not show any significant difference between each other, suggesting that antler side and aspect did not affect the MNT measurements. For MNT validation, the cut-off point was set at 50 newtons to minimise discomfort in animals and reduce tissue trauma; thereby, readings of the algometer that exceeded this value were labelled as “No Response” or “NR” to avoid any additional discomfort to the animals and any harm to the site of the stimulus. However, in the study for the assessment of the efficacy for articaine hydrochloride, any value of more than 50 N was considered 50 N. In the MNT validation study, animals that were recorded as “No Response” showed tonic immobility behaviour, which is an evolutionary adaptation for self-preservation and anticipation of any possible danger in nature [33,34]. In such cases, the hydraulic crush was opened, thereby releasing the immobilised deer and was gently re-closed in an attempt to reduce stress and make them more responsive to the pain assessment. Our experiment cut-off force (50 newtons) was approximately three-fold of the average threshold readings (~16 N), which also aligned with a previously published study by Carroll (1959), as cited in Le Bars et al. (2001), which determined the cut-off point as three-times the threshold of the controls to prevent tissue damage [8,35].

There was an observable acclimatisation to the MNT measurements in deer over the course of this study. Deer are naturally apprehensive and try to avoid handling about the antlers, by moving their heads. By the third day of the study, the enrolled deer stood quietly with predictable behaviour. Stress, anxiety, and diversions of attention are known to change the perception of noxious stimuli [36]. This reflects why the mechanical nociceptive thresholds must always be recorded with minimal restraint, and in a quiet area. In humans, skin bruising is reported when repeated measurements were applied at the same site over 3 consecutive days [37]. Therefore, in this study, we performed our experiment on three alternate days (every other day) to minimise tissue damage. Furthermore, the results of this study suggest that the pain threshold increases significantly on day three of the study compared to days 1 and 2. The frequency of animals with no response (due to tonic immobility) was less on the third day, as compared to the first two days. Additionally, only those measurements acquired on day three showed normal distribution compared to days one and two. This finding suggests a decrease in animal distress and reduced anticipation during the procedure, which could be due in part to the habituation of the animals to the handling facilities, personnel, and procedures, resulting in a more relaxed posture and less tonic immobility. These findings aligned with those of Stubsjøen et al. (2010), which showed that habituation is crucial in order to reduce the variability of mechanical nociceptive thresholds in animals [8]. Furthermore, gentle stroking of the head of the animal and slight occlusion of the vision by lightly cupping the hand near the ipsilateral eye, prior to the algometer placement, helped in obtaining consistent readings.

Some research studies investigating pain assessment showed a decrease in MNT when measured over consecutive days. Stubsjøen et al. (2010) demonstrated a decrease in MNT in sheep over three consecutive days, and Musk et al. (2017) found a decreased MNT in bull calves tested over six days [38]. In contrast, an increase in MNT measurements was observed in the current study. This could be species variation, or the procedure followed in this study did not cause any inflammation around the site of application, which might increase sensitivity to mechanical nociceptive thresholds. Therefore, it is important to validate the test and acclimatise animals prior to conducting pain assessment during clinical research studies, in order to decrease confounding factors.

The reliability of the assessments might be increased by introducing and re-exposing the animals to novel situations. During each assessment day, only one person recorded pain using algometry to decrease the chance of variability in the measurements. The algometer was applied at a regular interval to maintain consistency. It was observed that when the algometer was applied rapidly after initial touch, the deer reacted quickly in a more predictable manner. This could be explained by the sensory nerve pathways arising from the trigeminal nerve (CNV) in growing antlers and A-delta fibres, which are the receptors for pressure and which transmit pain information from an acute noxious stimulus [39,40]. Alternatively, if the algometer is applied slowly, it is more likely that the reaction would be due to A-beta fibres that transmit information from skin receptors and C fibres, which are slow pain receptors [39,40].

The MNT testing was performed at four sites around the base of the antlers; cranial, medical, caudal and lateral, on each of the left and right antlers. The results demonstrated a non-significant difference between the left and right antlers, which indicate that these could be treated as repeatable subjects in future studies. MNT results from all four different sites were normally distributed in the case of cranial, medial and lateral locations; however, the caudal site was not normally distributed. This could be due to the topographical location and acute angle of the developing antler (Figure 2), which limits access for the perpendicular placement of the algometer tip, compared to the other sites.

A positive correlation was demonstrated between animal body weight and MNT, between antler length and MNT, and between animal body weight and antler length, which means that, the heavier yearling deer had longer antlers. These results suggest that larger animals with increased body weight or animals with increased antler length are more tolerant of a painful stimulus applied to the velvet. It is likely that the increased tolerance could be a result of tissue changes within the antler as it develops. For example, the network of sensory nerve fibres in the vascular layer and those penetrating the superficial layers of the dermis might atrophy as the antler matures, and this process might begin much earlier than the point at which the antler is entirely calcified and cast. Further studies are required to establish when the nerve network to the velvet tissue declines, and if the hardening of the underlying cartilaginous tissue affects this process. It was already documented that the most proximal region of the antler is more calcified compared to the distal tip [41]. Consequently, it could be surmised that the longer and thus, more developed antlers in this cohort would have had increased pain tolerance at the base. Furthermore, it was demonstrated that during the growth phase, male deer have virtually undetectable plasma testosterone (T) hormone levels, which might influence behaviour, making them more docile and less likely to damage the growing tissue [42].

Various authors suggested the use of articaine hydrochloride for local analgesia for human adults [43] and infants [44], and animals such as rats [45], cattle [46,47], and deer [26,27]. Therefore, the increasing demand for this local anaesthetic, its safety and rapid onset, encouraged us to investigate its duration of action on red deer, which is poorly studied. Currently, in New Zealand, the only licensed local anaesthetic used in deer is lignocaine hydrochloride [20,21], which itself is poorly studied in terms of pharmacokinetics and using more objective pain assessments such as MNT. The authors previously, only depended on behavioural analysis [23,48], and electrical stimulation [49] to prove the pain relief effects of lignocaine in deer. Our study in combination with the findings from two studies by Venkatachalam et al. [26,27] suggests that articaine hydrochloride can be a better candidate for pain relief for deer, after velvet antler removal, since it has a rapid onset of action, it is safer than lignocaine and bupivacaine, it has an analgesic duration of up to 6 h, and shows minimal residues after application. The study was stopped after 6 h, as the recorded force readings reached close to 20 N, which was the boundary of the baseline category (according to our established scaling system in force reading) (see Table 1). As a result of recent studies, which also includes the pharmacokinetic findings, its application in deer now surpasses that of lignocaine hydrochloride, which was used as the only licensed product for deer for two decades. 

The primary limitation faced in this study was managing the deer’s innate behaviour. Deer are a prey species that display a particular unlearned behaviour known as tonic immobility when faced with a threat. Tonic immobility is a behaviour expressed as a last resort, most typically when a predator is in close proximity or has made contact. This should be distinguished from freezing behaviour that occurs when a prey animal is under pursuit and is attempting to camouflage within its environment [50]. Tonic immobility can occur during periods of physical restraint, and these events might last from seconds to hours and can persist after the animal is physically released. Signs of tonic immobility include bradycardia, bradypnoea, salivation, defecation, and urination, which is a direct result of parasympathetic activity [51]. The mechanism behind this phenomenon is a result of the complex interplay between serotonin, the stress hormone corticotropin-releasing factor (CRF) and dopamine with the amygdala, and varies between species [51]. Over the course of this trial, non-resolving tonic immobility was encountered, whilst the deer were physically restrained in the hydraulic crush, occurring in 14 out of 960 measurements. These measurements were recorded as NR, indicating no response, and an algometer reading beyond the predetermined cut-off point (50 N). Habituation to the hydraulic crush, holding pens, and experimental area occurred periodically during the months prior, as the facility was used for other animal husbandry procedures such as anthelmintic drenching and vaccinations. However, animal handlers and the pain assessor could be viewed as a threat by the deer. The removal of antlers without veterinary supervision or anaesthesia is considered a contravention of the Code of Recommendations and Minimum Standards for the Welfare of Deer During the Removal of Antlers (1992) [52], and Animal Welfare Act 1999 [53], and is accordingly a prosecutable criminal offence in New Zealand. It is for this reason a control group receiving no analgesia was not included in this study to compare against the articaine treatment group.

In this study, a hand-held algometer was used to measure the MNT. This device was successfully used in other species, including humans. The limitations to the use of this device include variability in the rate of application of pressure on the tip and suitability of the tip diameter for the size of the animal. The assessor applied the algometer for approximately 1 s, with the cessation of pressure occurring when the deer moved away from the algometer tip. An additional improvement would be a remotely controlled algometer that can apply pressure at a consistent speed. This study provides a basis for MNT testing of growing deer antlers, and additional investigations and development of standardised testing protocols would allow a comparison between studies. In terms of the articaine study, the current experiment only investigated the effect of this local anaesthetic for a peri-operative pain management. There might be scope to use articaine hydrochloride in a formulation that prides post-operative analgesia with an extended duration of effect.

## 5. Conclusions 

This study validates the use of MNT in red deer, as a method of testing the efficacy of analgesia for antler removal. A number of confounding factors were determined, which could alter the level of tolerance to pain in deer undergoing MNT, such as body weight and antler length. To our knowledge, this is the first study on MNT in red deer antlers and forms the basis for future research. The results from the articaine trial suggests that this local anaesthetic is an ideal alternative for the practice of antler removal due to its rapid onset, suitable duration of pain relief, wide safety margins, and high elimination from the body.

## Figures and Tables

**Table 1:** The least-squares means (±SEM) of MNT (N) for algometry validation and articaine study. The values below depict MNT disregarding antler side and site. N = 40 animals for the validation study, and 10 for articaine study.

Time	Pressure Algometry Validation			Articaine Study
	Day 1	Day 2	Day 3	
Baseline	15.49 ± 0.55 ^a^ (B)	14.18 ± 0.55 ^b^ (B)	18.74 ± 0.55 ^c^ (B)	15.74 ± 0.95 * (B)
10 min				49.37 ± 0.95 * (H)
2 h				41.45 ± 0.95 * (H)
4 h				26.46 ± 0.95 * (L)
6 h				21.55 ± 0.95 * (L)

* statistically significant difference, ^a–c^ statistically significant difference. B = baseline force, H = High force, M = Medium force, L = Low force.
